# Association of gut microbiota with critical pneumonia: A two-sample Mendelian randomization study

**DOI:** 10.1097/MD.0000000000039677

**Published:** 2024-10-18

**Authors:** Yuanxiao Li, Mengru Fang, Dan Li, Peirun Wu, Xuan Wu, Xiaonan Xu, Hanwei Ma, Yan Li, Ni Zhang

**Affiliations:** a Department of Pediatric Gastroenterology, The Second Hospital, Lanzhou University, Lanzhou, Gansu Province, China; b The Second Clinical Medical School, Lanzhou University, Lanzhou, Gansu Province, China.

**Keywords:** critical pneumonia, genetic variants, gut microbiota, Mendelian randomization

## Abstract

This study investigated the causal effect of gut microbiota on critical pneumonia. Data came from a large-scale gut microbiota data set (n = 18,340) and the critical pneumonia genome-wide genotyping array (cases n = 2758 and controls n = 42,8607). Inverse variance weighting was used as the primary Mendelian randomization (MR) analysis method. Weighted median, MR-Egger, simple model, weighted model, and MR-Egger, were used to evaluate robustness. Sensitivity analysis used Cochran Q test, MR-Egger intercept test, and MR-PRESSO. For critical pneumonia, inverse variance weighting estimates suggested that Class Verrucomicrobiae (OR = 0.415; 95% CI: 0.207, 0.833; *P* = .013), Family Verrucomicrobiaceae (OR = 0.415; 95% CI: 0.207, 0.833; *P* = .013), Genus Akkermansia (OR = 0.415; 95% CI: 0.207, 0.833; *P* = .013), Genus LachnospiraceaeFCS020group (OR = 0.449; 95% CI: 0.230, 0.890; *P* = .021), Genus Parasutterella (OR = 0.466; 95% CI: 0.233, 0.929; *P* = .030), Genus Prevotella7 (OR = 0.645; 95% CI: 0.432, 0.960; *P* = .031), Order Verrucomicrobiales (OR = 0.415; 95% CI: 0.207, 0.833; *P* = .013), and Phylum Cyanobacteria (OR = 0.510; 95% CI: 0.272, 0.956; *P* = .036) had a reduced risk, while Family Enterobacteriaceae (OR = 2.746; 95% CI: 1.008, 7.474; *P* = .048), Genus RuminococcaceaeUCG003 (OR = 2.811; 95% CI: 1.349, 5.851; *P* = .006) and Order Enterobacteriales (OR = 2.746; 95% CI: 1.008, 7.474; *P* = .048) were associated with an increased risk. Sensitivity analyses confirmed that the aforementioned correlations were robust.

## 1. Introduction

Due to the multitude of other factors and microbiota that can cause clinical decline in critically ill patients who are complex, timely diagnosis of hospital-acquired pneumonia and ventilator-associated pneumonia is particularly difficult in the intensive care unit. These conditions result in significant in-hospital morbidity and mortality.^[1]^ Further study on critical pneumonia is desperately needed since early commencement of appropriate antimicrobial therapy and proper identification of critical pneumonia are vital to improve the survival percentage of critically ill patients.^[2]^

The origin and course of numerous diseases are intimately linked to the gut microbiome, a diverse collection of microorganisms that inhabits the digestive tracts of both humans and animals.^[3]^ Pneumonia is avoided by a healthy upper respiratory tract and gut microbiota because they inhibit the growth of pathogenic bacteria and control the immune system. Intensive care techniques and antibiotic therapies, however, can upset the microbiota, reducing its ability to function and increasing the risk of pneumonia.^[4]^ TaoZou et al discovered that alterations in the fecal microbiota were connected to fecal SARS-CoV-2 levels and COVID-19 severity in comparison to controls in a research including 15 COVID-19 patients.^[5]^ Techniques that modify the gut microbiota could lessen the disease’s severity. A reduction in risk was observed for Gammaproteobacteria (odd than [OR] = 0.94, 95% confidence interval [CI], 0.89 0.99, *P* = .0295) and streptococcus (OR = 0.95, 95% CI, 0.92 to 1.00, *P* = .0287), while Subdoligranulum (OR = 0.80, 95% CI, 0.69–0.92, *P* = .0018) showed a negative correlation with COVID-19 severity. The strong correlation between cyanobacteria (OR = 0.85, 95% CI, 0.76–0.96, *P* = .0062), Lactobacillales (OR = 0.87, 95% CI, 0.76–0.98, *P* = .0260), and Christensenellaceae (OR = 0.87, 95% CI, 0.77–0.99, *P* = .0384) was validated by sensitivity analysis. These results provide fresh light on the mechanisms behind the gut microbiota-mediated development of COVID-19 and imply that the gut microbiota may affect the susceptibility and severity of the virus in a causative manner.^[6]^ More concrete data is required in this area as the association between the gut microbiota and critical pneumonia is not well established due to the paucity of studies conducted to date.

Thus, it is essential to comprehend the relationship between the gut microbiota and critical pneumonia in order to develop strategies for easing suffering and enhancing prognoses for these patients. Nevertheless, more research is necessary to fully understand the intricate link between critical pneumonia and the gut flora. We employed a two-sample Mendelian randomization (MR) approach to investigate the bidirectional causal link between the gut microbiota and critical pneumonia.^[7]^ Should thoroughly investigate the possibility that the gut microbiota’s makeup influences the severity and vulnerability to critical pneumonia. By controlling gut microbiota, the findings of this study may offer novel approaches to the tailored treatment of critical pneumonia.

## 2. Methods

### 2.1. Study design

**Figure 1.**
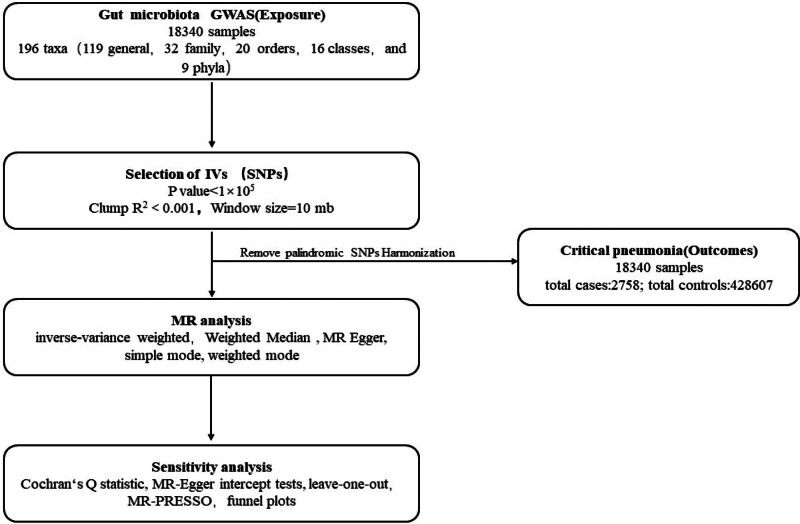
Overview of the current Mendelian randomization study.

The genome-wide association study (GWAS) summary statistics were used to perform two-sample MR analyses to estimate the causal effects of gut microbiota on critical pneumonia. IVs were selected to fulfill 3 assumptions: IVs must be strongly associated with the exposures of interest; IVs were independent of unmeasured confounders; and IVs were related to outcomes only through the exposure of interest, not through confounders.^[8]^ Gut microbiota and critical pneumonia served as exposure and outcome, respectively, in the MR analyses. A flowchart depicting the procedure can be found in Figure 1.

### 2.2. Gut microbiota samples

Summary statistics for the composition of the human gut microbiome are from a large-scale multiethnic GWAS meta-analysis comprising 24 cohorts (18,340 participants).^[9]^ Included cohorts were conducted in the United States, Canada, Israel, South Korea, Germany, Denmark, the Netherlands, Belgium, Sweden, Finland, and the United Kingdom. Twenty cohorts included samples with only 1 ancestry, and most participants were of European ancestry (16 cohorts, n = 13,266). A total of 211 taxa (131 genera, 35 families, 20 orders, 16 classes, and 9 phyla) were included. Of these, 12 were unknown genera and 3 were unknown families. These taxa were therefore excluded from the study, leaving 196 taxa for subsequent analyses. The included cohorts were adjusted for the covariates sex and age in the calculations. The summarized statistics of the association study are publicly available on the website https://mibiogen.gcc.rug.nl.

### 2.3. Critical pneumonia samples

Summary statistics for critical pneumonia phenotypes were obtained from the latest version of the genome-wide critical pneumonia genotyping array, which contains a total of 2758 cases and 428,607 controls.^[10]^ Detailed information for genome-wide association studies were presented in Table S1, Supplemental Digital Content, http://links.lww.com/MD/N552.

### 2.4. Selection of IVs

The bacterial taxa were analyzed at 5 levels (phylum, class, order, family, and genus). Because genetic loci for the gut microbiota identified by GWAS rarely reach genome-wide significance levels (*P* < 5 × 10^‐8^) and the genetic variants used for MR must be representative of the microbiome, suggestive *P*-values of <1 × 10^‐5^ were used, as in previous MR studies.^[11]^ To obtain independent single nucleotide polymorphisms (SNPs), linkage disequilibrium clumping with a clumping window of 10 mb was applied, and SNPs with large *P*-values at a threshold of linkage disequilibrium *R*^2^ ≥ 0.001 were eliminated, using the European population as a reference. The F-statistic was calculated to quantify the strength of genetic variation, and SNPs with an F-statistic >10, indicating insufficient strength,^[12]^ were discarded. Proxies with a cutoff value of *R*^2^ > 0.8 were identified for the missing SNPs in the resulting GWAS dataset (https://snipa.org/snipa3/). If no suitable proxy was available, the SNPs were discarded. To avoid strand direction bias or allele coding, palindromic SNPs were removed. During harmonization, alleles were aligned to the human genome reference sequence (build 37) and ambiguous and duplicate SNPs were removed. Genome-wide significant and independent SNPs that were used as instruments for gut microbiota were respectively presented in Table S2, Supplemental Digital Content, http://links.lww.com/MD/N552.

### 2.5. Statistical analyses

We estimated the causal relationships between 196 gut microbiota and critical pneumonia separately using different MR methods. To detect the causal relationships between exposure (gut microbiota) and outcomes (critical pneumonia), inverse variance weighting (IVW) method was used as the primary MR analysis method. The IVW method, an extension of the Wald estimator for estimating causal effects, restricts the intercept to 0. It calculates the total causal effect using a weighted linear regression model with the weighting coefficient.^[13]^ To assess the robustness of our study, we also applied several other MR methods, including WM, MR-Egger, simple mode, weighted mode, and MR-Egger. Cochran IVW Q-statistics and leave-one-out analyses were used to assess potential heterogeneity. The MR-Egger intercept and MR pleiotropy residual sum and outlier test (MR-PRESSO) were performed to determine whether the results of the MR analyses were affected by directional horizontal pleiotropy.^[14,15]^ All MR analyses were performed with the packages “TwoSampleMR” and “MRPRESSO” in the R software.

## 3. Results

**Figure 2.**
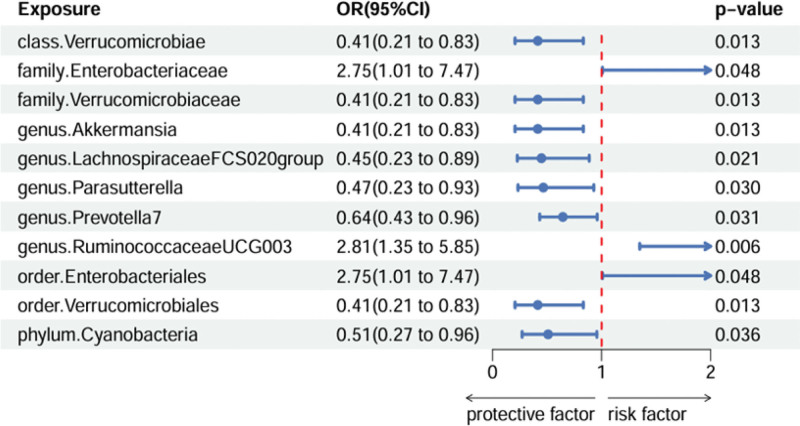
Gut microbiota which associated with critical pneumonia.

At the class level, by using the IVW method, we found a suggestive causal association of increase in Verrucomicrobiae (OR = 0.415; 95% CI: 0.207, 0.833; *P* = .013) and lower risk of critical pneumonia. At the family level, by using the IVW method, we found a suggestive causal association of increase in Enterobacteriaceae (OR = 2.746; 95% CI: 1.008, 7.474; *P* = .048) and higher risk of critical pneumonia, while genetically increased in Verrucomicrobiaceae (OR = 0.415; 95% CI: 0.207, 0.833; *P* = .013) was related to protective effects on critical pneumonia. At the genus level, by using the IVW method, we found a suggestive causal association of increase in RuminococcaceaeUCG003 (OR = 2.811; 95% CI: 1.349, 5.851; *P* = .006) and higher risk of critical pneumonia, while genetically increased in Akkermansia (OR = 0.415; 95% CI: 0.207, 0.833; *P* = .013), LachnospiraceaeFCS020group (OR = 0.449; 95% CI: 0.230, 0.890; *P* = .021), Parasutterella (OR = 0.466; 95% CI: 0.233, 0.929; *P* = .030) and Prevotella7 (OR = 0.645; 95% CI: 0.432, 0.960; *P* = .031) were related to protective effects on critical pneumonia. At the order level, by using the IVW method, we found a suggestive causal association of increase in Enterobacteriales (OR = 2.746; 95% CI: 1.008, 7.474; *P* = .048) and higher risk of critical pneumonia, while genetically increased in Verrucomicrobiales (OR = 0.415; 95% CI: 0.207, 0.833; *P* = .013) were related to protective effects on critical pneumonia. At the phylum level, by using the IVW method, we found a suggestive causal association of increase in Cyanobacteria (OR = 0.510; 95% CI: 0.272, 0.956; *P* = .036) and lower risk of critical pneumonia (Fig. 2). The scatter plots and forest plots for the causal relationship between gut microbiota and critical pneumonia were respectively presented in Figure S1, Supplemental Digital Content, http://links.lww.com/MD/N551 and Figure S4, Supplemental Digital Content, http://links.lww.com/MD/N551. MR results from gut microbiota to critical pneumonia in discovery dataset were presented in Table S3, Supplemental Digital Content, http://links.lww.com/MD/N552.

Cochran Q statistics showed no significant heterogeneity in selected IVs (*P* > .05 in IVW and MR-Egger methods). The funnel plots for the causal relationship between gut microbiota and critical pneumonia were presented in Supplementary Figure S2, Supplemental Digital Content, http://links.lww.com/MD/N551. Both the MR-Egger intercept and the MR-PRESSO global test confirmed there is no significant directional horizontal pleiotropy (*P* > .05). Additionally, the leave-one-out analysis revealed that there are no outlier IVs that would have a significant impact on the result if retained. The leave-one-out plots for the causal association between gut microbiota and critical pneumonia were presented in Figure S3, Supplemental Digital Content, http://links.lww.com/MD/N551.

## 4. Discussion

To the best of our knowledge, this is the first study to investigate the possible link between critical pneumonia and gut microbiota using MR analysis. This study demonstrates that 11 gut microbiota are linked with critical pneumonia using large-scale aggregated statistics of gut microbiome GWAS and critical pneumonia genome-wide gene arrays. emphasizing the connection between respiratory illnesses and the gut–lung axis.^[16,17]^

Trillions of bacteria inhabit the gastrointestinal tract, making up the gut microbiome. The gut microbiota is made up of over a 1000 different species of bacteria, 90% of which are classified into 4 phyla: actinobacteria, proteobacteria, firmicutes, and Bacteroidetes.^[18]^ According to certain observational studies, disruption of the gut microbiota frequently coexists with critical pneumonia. For instance, even after the illness had cleared up, the gut microbiota of COVID-19 patients still had abnormalities in the production of L-isoleucine and short-chain fatty acids (SCFA). The pathophysiology and outcome of SARS-CoV-2 infection are significantly influenced by the function of gut microorganisms, as evidenced by the correlation between these bacteria’ activity and the host immune response.^[19]^ Moreover, it was demonstrated that a varied gut microbiome can avert mortality in a mouse model of pneumococcal pneumonia.^[20]^ Asthma, chronic obstructive pulmonary disease (COPD), and chronic bronchitis are examples of chronic lower respiratory diseases that may be exacerbated by microbial products’ steady-state tetanic signaling of pattern recognition receptors, as demonstrated by Jeremy P. McKee et al.

We employed various MR methods to estimate the causal association between gut microbiota and critical pneumonia in a cohort of 196 individuals. Ultimately, our analysis revealed that 11 bacterial groups exhibited significant correlations with the severity of critical pneumonia. To ensure the robustness of our findings, we conducted a comprehensive set of sensitivity analyses including MR analysis, MR-Egger regression, WM, Cochrane Q test, and MR-Egger intercept test. The presence of 2 bacterial groups, Verrucomicrobiae (OR = 0.415; 95% CI: 0.207, 0.833; *P* = .013) and Cyanobacteria (OR = 0.510; 95% CI: 0.272, 0.956; *P* = .036), was associated with a reduced risk of critical pneumonia. Two other bacterial groups, Enterobacteriaceae (OR = 2.746; 95% CI: 1.008, 7.474; *P* = .048) and RuminococcaceaeUCG003 (OR = 2.811; 95 % CI: 1.349, 5.851; *P* = .006), were found to be causally linked to an increased risk of critical pneumonia. The study also revealed that the abundance of 7 specific microbiota species was associated with protection against critical pulmonary inflammation: Verrucomicrobiaceae (OR = 0.415; 95% CI: 0.207, 0.833; *P* = .013), Akkermansia (OR = 0.415; 95% CI: 0.207, 0.833; *P* = .013), LachnospiraceaeFCS020group (OR = 0.449; 95% Cl: 0.230, 0.890; *P* = .021), Parasutterella (OR = 0.466; 95% CI: 0.233, 0.929; *P* = .030), and Prevotella7 (OR = 0.645; 95% CI: 0.432, 0.960; *P* = .031). Furthermore, it was observed that Cyanobacteria played a protective role in reducing the risk of critical pneumonia due to their ability to produce various metabolites such as polysulfates and lectins which possess potent antiviral activity and immune-enhancing effects.^[21]^

Currently, there are studies indicating that patients with critical pneumonia often exhibit disturbances in their intestinal microbiota; however, the research findings remain inconclusive and require further investigation. The primary strength of this study lies in its utilization of the MR method, which mitigates confounding factors and reverse causality that may influence the results. Nevertheless, certain limitations exist within this study. Firstly, the small sample size and inadequate gut microbiome GWAS capacity may result in insufficient identification of specific bacterial characteristics at a genetic level. Therefore, it is anticipated that analysis at a higher classification level will be conducted. Once gut microbiome GWAS incorporates an adequate sample size, these more precise features can be identified with greater resolution. Secondly, due to the absence of demographic statistics (such as gender and ethnicity) in previous investigations on critical pneumonia,^[22]^ separate gender analysis was not performed in this study potentially impacting our results. Future work would benefit from conducting sex-specific MR analysis. The sample size of the gut microbiome was relatively small, and no reverse MR analysis was conducted due to concerns about potential weak instrumental bias in the results. Additionally, it is important to note that the majority of included cohorts were of European descent, thus caution should be exercised when generalizing the findings to other ethnic groups. Consequently, further investigation involving diverse ethnic populations is warranted.

In conclusion, this MR study suggests a potential causal relationship between the gut microbiota and the risk of critical pneumonia. The specific composition of intestinal bacteria identified in this study may modulate the occurrence and progression of critical pneumonia, thereby offering promising avenues for its prevention and treatment.

## Acknowledgments

We thank all the authors and participants of the GWAS. This study was supported by the Lanzhou Chengguan District Science and Technology Planning Project (No. 2020SHFZ0041), the Cuiying Scientific Training Program for Undergraduates of Lanzhou University Second Hospital (No. CYXZ2023-03, CYXZ2023-33), Cuiying Scientific and Technological Innovation Program of The Second Hospital & Clinical Medical School, Lanzhou University (CY2023-BJ-10).

## Author contributions

**Conceptualization:** Yuanxiao Li, Mengru Fang, Dan Li, Peirun Wu, Xuan Wu, Xiaonan Xu, Hanwei Ma.

**Data curation:** Yuanxiao Li, Dan Li, Ni Zhang.

**Formal analysis:** Yuanxiao Li, Peirun Wu, Yan Li, Ni Zhang.

**Funding acquisition:** Yuanxiao Li, Mengru Fang, Ni Zhang.

**Investigation:** Yuanxiao Li, Mengru Fang, Xuan Wu.

**Methodology:** Yuanxiao Li, Xiaonan Xu.

**Project administration:** Mengru Fang, Ni Zhang.

**Resources:** Yuanxiao Li, Hanwei Ma, Yan Li, Ni Zhang.

**Software:** Yuanxiao Li, Dan Li.

**Supervision:** Mengru Fang, Xuan Wu, Xiaonan Xu, Ni Zhang.

**Validation:** Mengru Fang, Peirun Wu, Ni Zhang.

**Visualization:** Peirun Wu.

**Writing – original draft:** Yuanxiao Li, Mengru Fang, Dan Li, Ni Zhang.

**Writing – review & editing:** Yuanxiao Li, Mengru Fang, Dan Li, Xuan Wu, Ni Zhang.

## Supplementary Material


