# Supplementary material for: Association of gut microbiota with critical pneumonia: A two-sample Mendelian randomization study
Source: Medicine (Baltimore). 2024 Oct 18;103(42):e39677. doi: 10.1097/MD.0000000000039677 (PMC11495696; doi:10.1097/MD.0000000000039677)

**Figure S1.** Scatter plots for the causal relationship between gut microbiota and critical pneumonia..

**Verrucomicrobiae.id.4029** **family.Enterobacteriaceae.id.3469** **family.Verrucomicrobiaceae.id.4036**

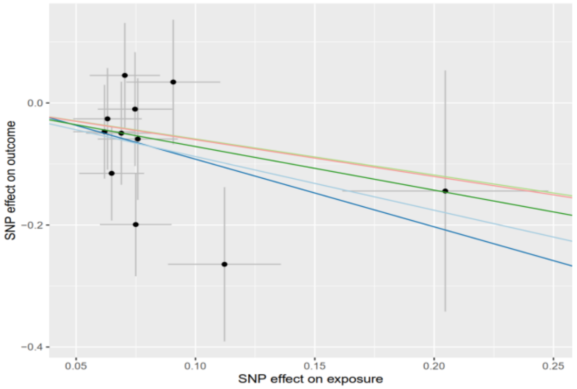


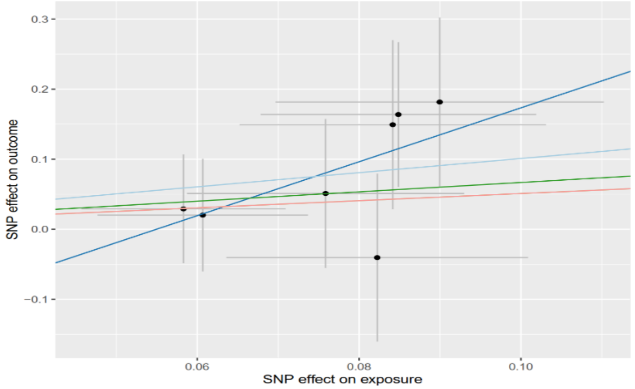


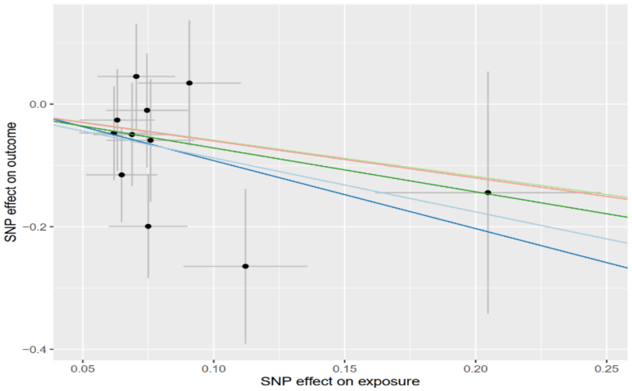


**genus.Akkermansia.id.4037** **genus.LachnospiraceaeFCS020group.id.11314** **genus.Parasutterella.id.2892**

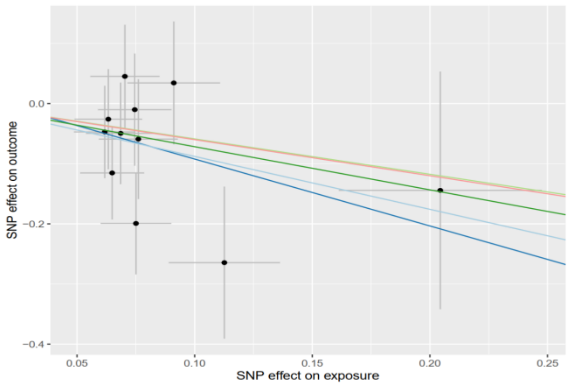


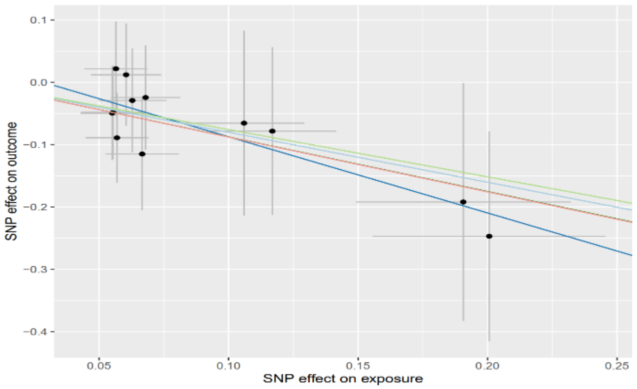


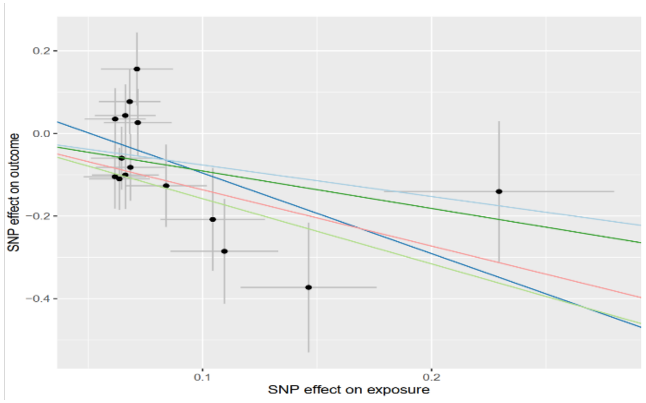


**genus.Prevotella7.id.11182** **genus.RuminococcaceaeUCG003.id.11361** **order.Enterobacteriales.id.3468**

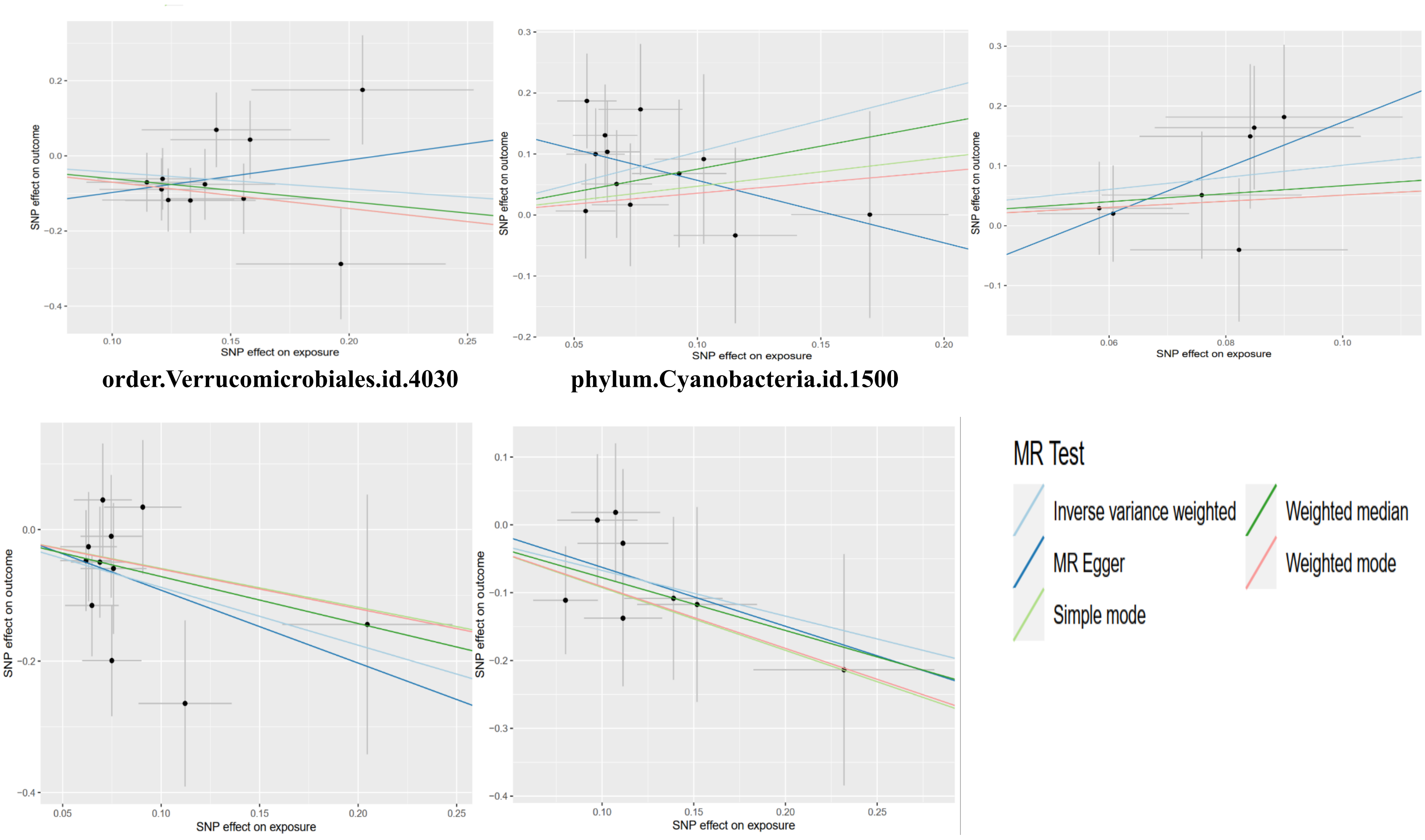

SNP effect on outcome
SNP effect on exposure
order.Verrucomicrobiales.id.4030
phylum.Cyanobacteria.id.1500
MR Test
Inverse variance weighted
MR Egger
Simple mode
Weighted median
Weighted mode

**Figure S2.** Funnel plots for the causal relationship between gut microbiota and critical pneumonia.

**Verrucomicrobiae.id.4029** **family.Enterobacteriaceae.id.3469** **family.Verrucomicrobiaceae.id.4036**

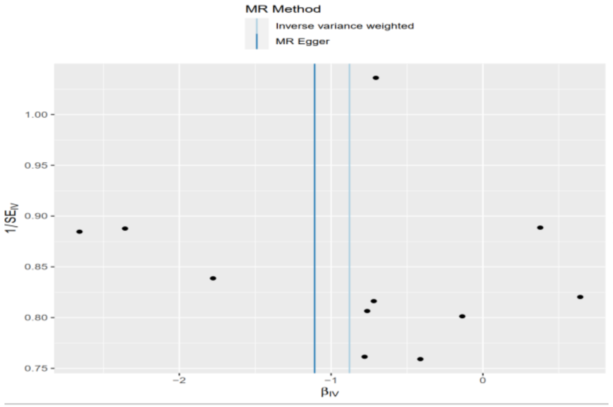


**genus.Akkermansia.id.4037**

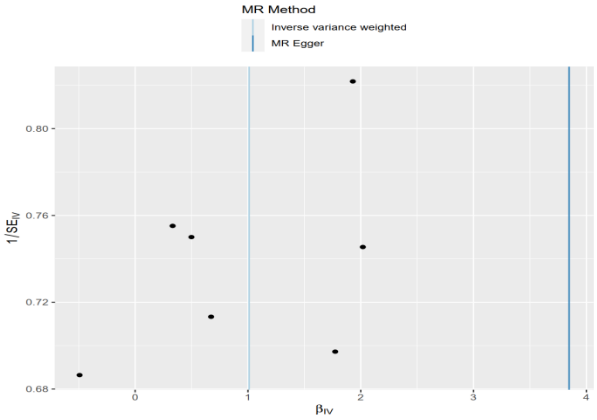


**genus.LachnospiraceaeFCS020group.id.11314**

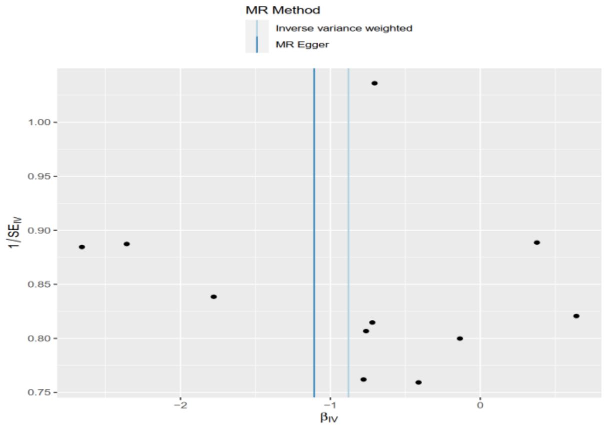


**genus.Parasutterella.id.2892**

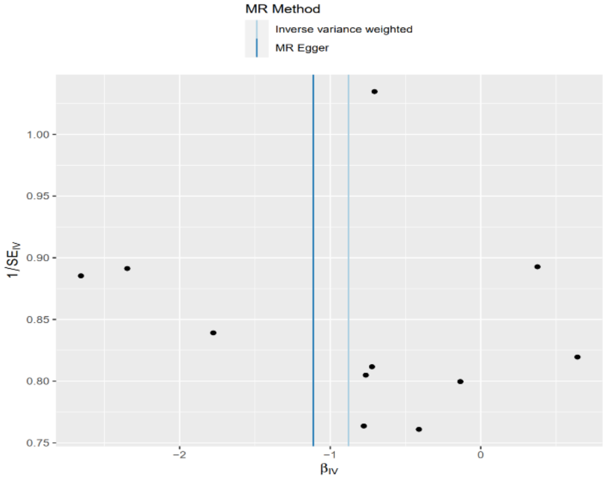


**genus.Prevotella7.id.11182**

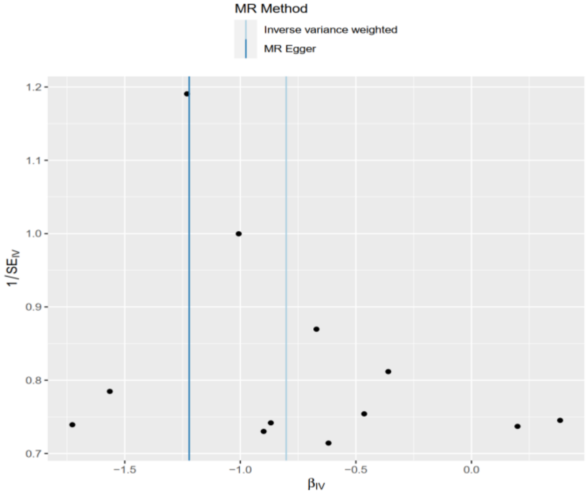


**genus.RuminococcaceaeUCG003.id.11361**

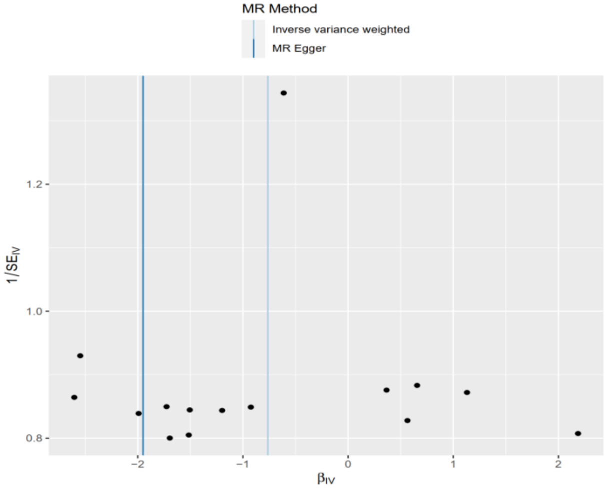


**order.Enterobacteriales.id.3468**

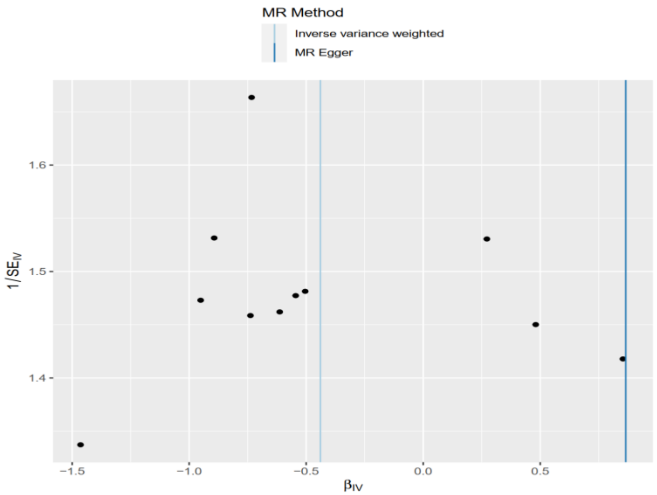


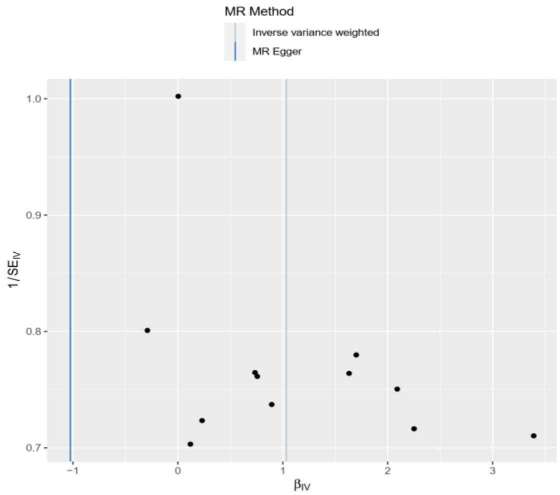


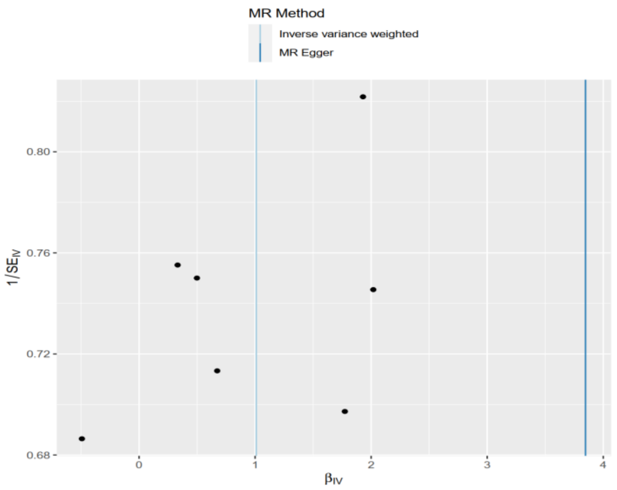


order.Verrucomicrobiales.id.4030

phylum.Cyanobacteria.id.1500

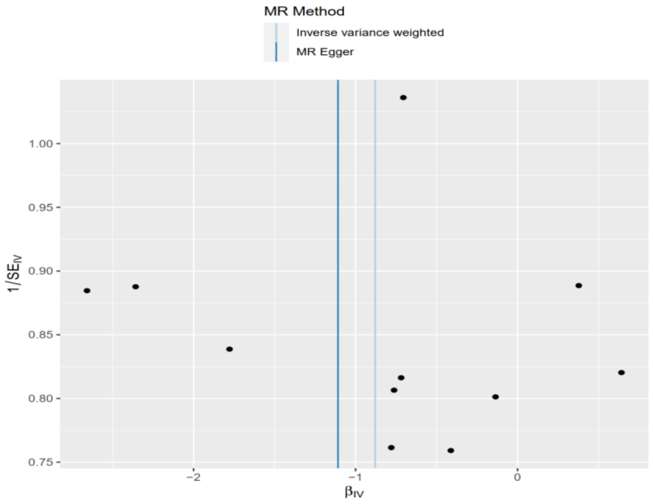


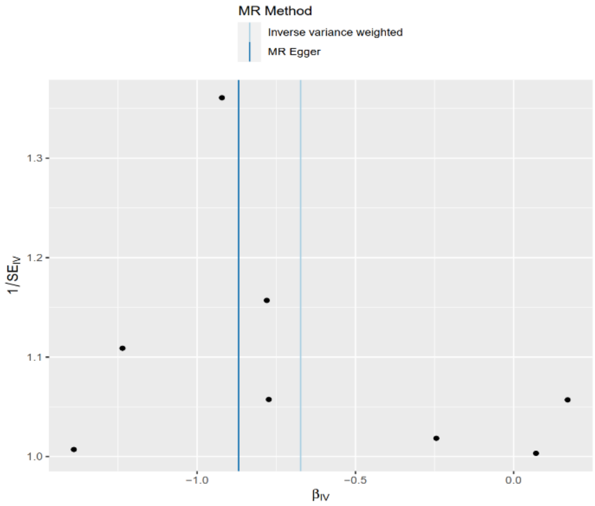

**Figure S3.** Leave-one-out plots for the causal association between gut microbiota and critical pneumonia.

**Verrucomicrobiae.id.4029** **family.Enterobacteriaceae.id.3469** **family.Verrucomicrobiaceae.id.4036**

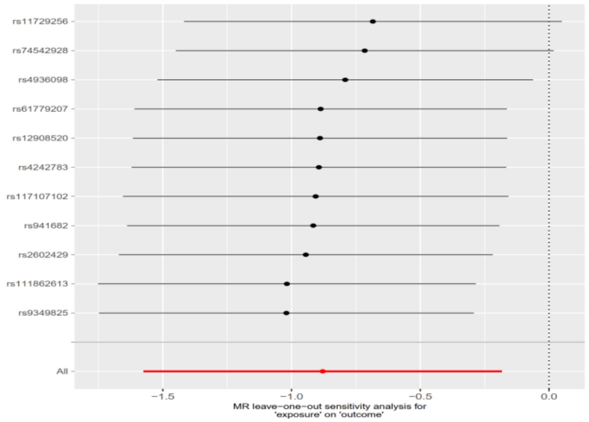


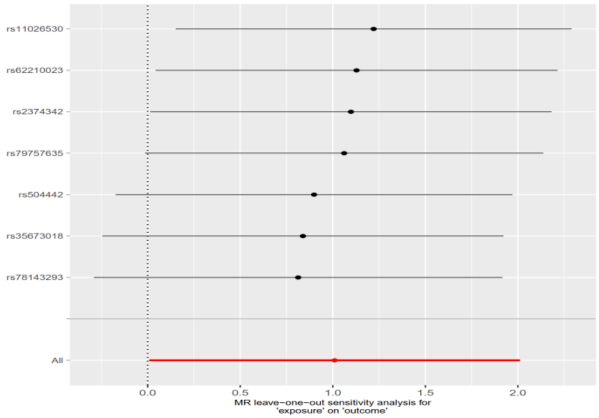


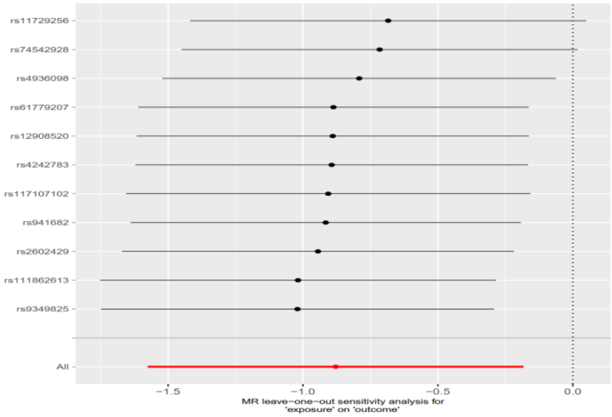


**genus.Akkermansia.id.4037** **genus.LachnospiraceaeFCS020group.id.11314** **genus.Parasutterella.id.2892**

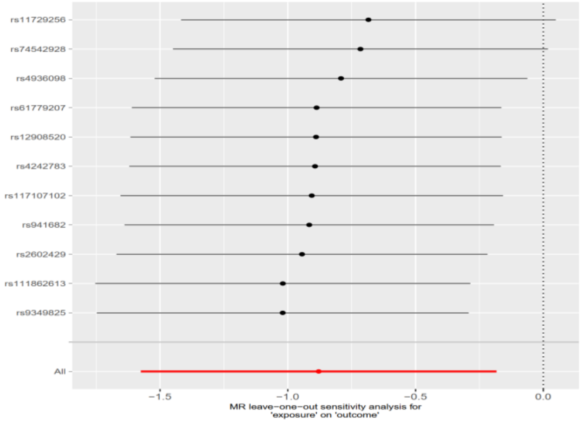


genus.Prevotella7.id.11182

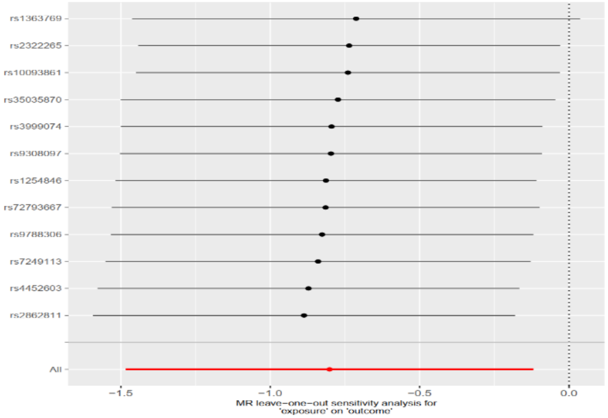


genus.RuminococcaceaeUCG003.id.11361

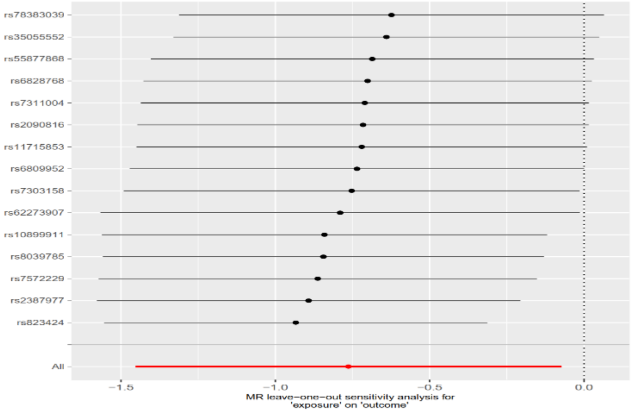


order.Enterobacteriales.id.3468

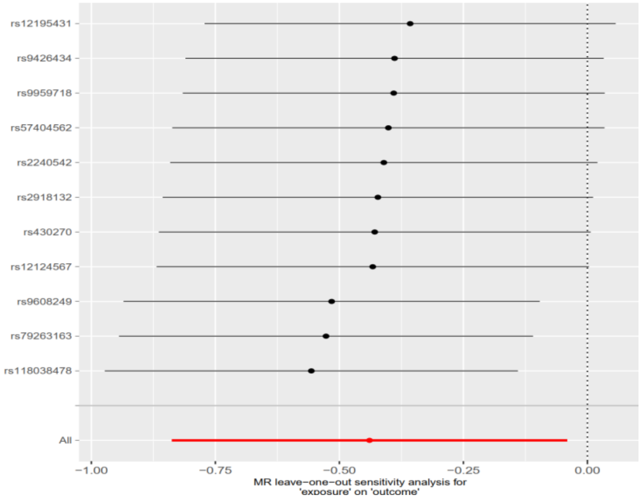


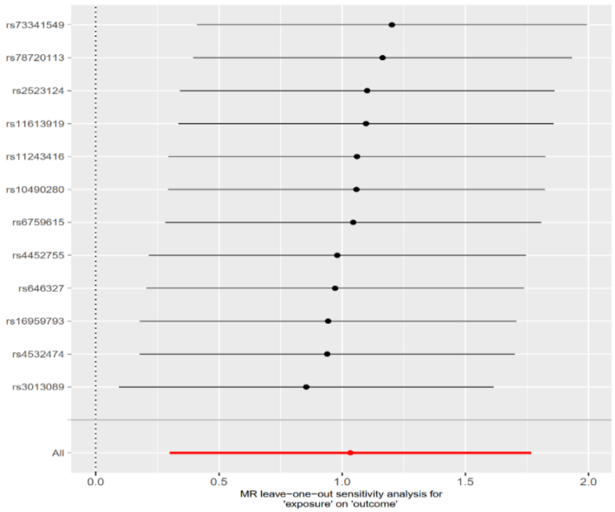


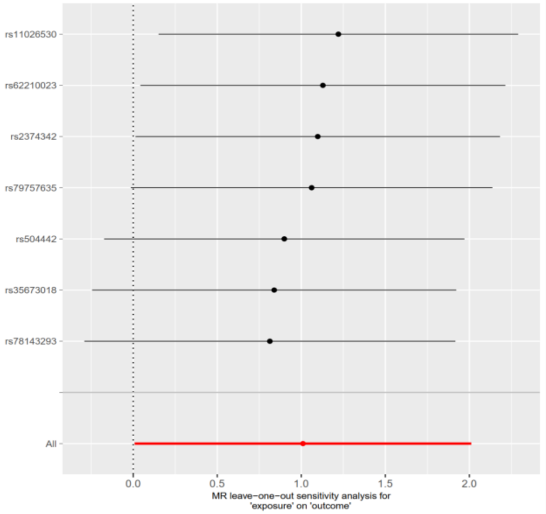

**order.Verrucomicrobiales.id.4030**

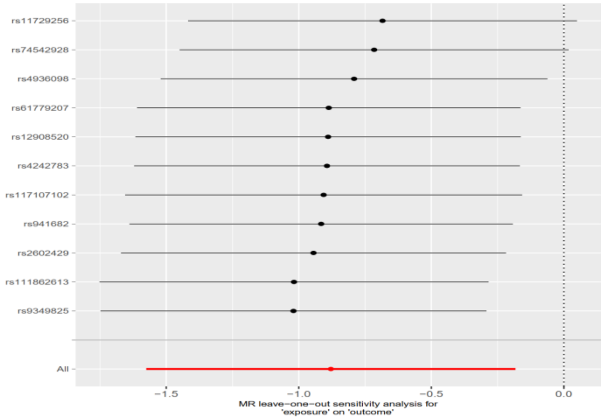


**phylum.Cyanobacteria.id.1500**

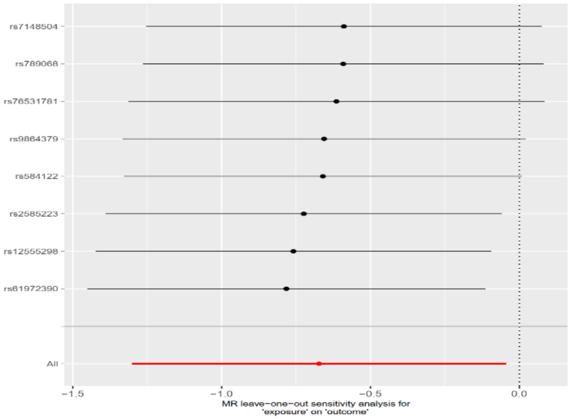


**Figure S4.** Forest plots for the causal association between gut microbiota and critical pneumonia

**Verrucomicrobiae.id.4029** **family.Enterobacteriaceae.id.3469** **family.Verrucomicrobiaceae.id.4036**

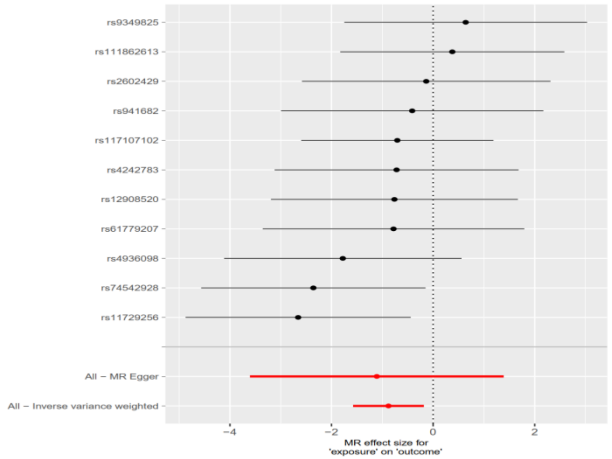


genus.Akkermansia.id.4037

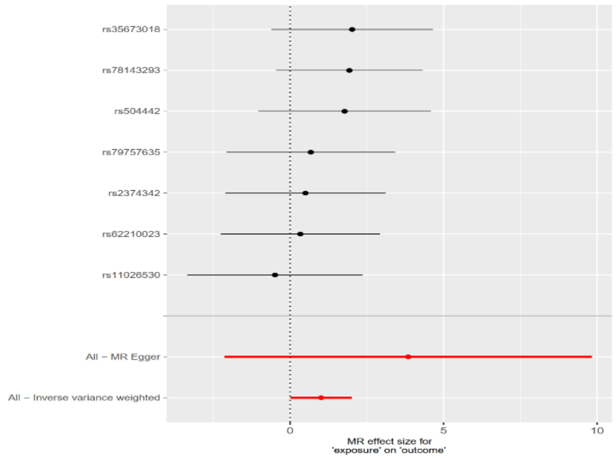


genus.LachnospiraceaeFCS020group.id.11314

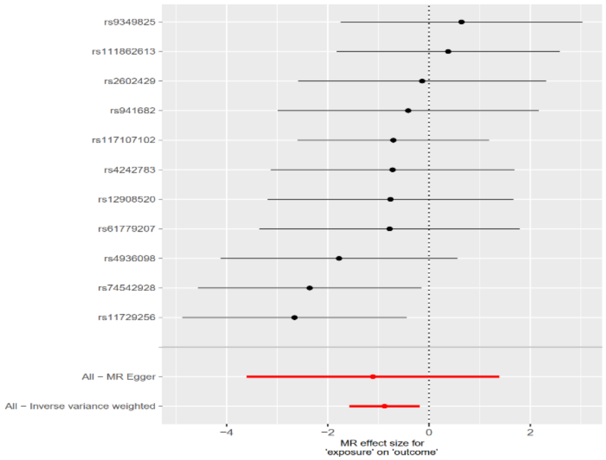


genus.Parasutterella.id.2892

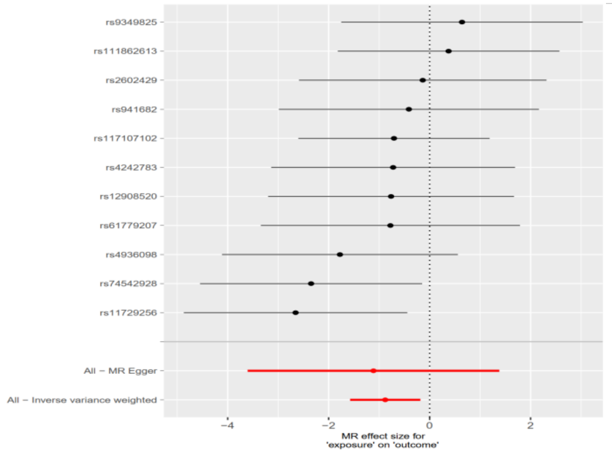


genus.Prevotella7.id.11182

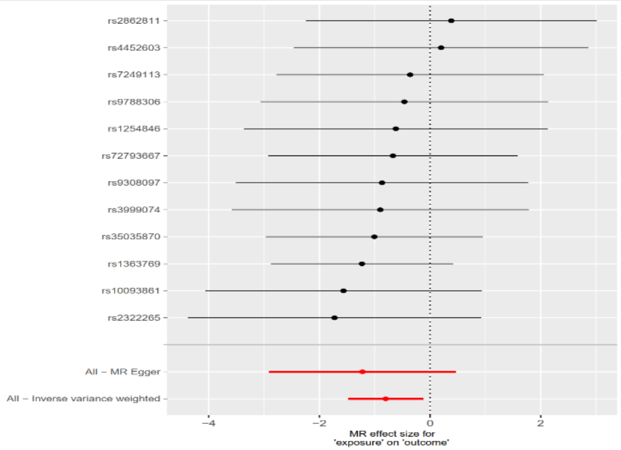


genus.RuminococcaceaeUCG003.id.11361

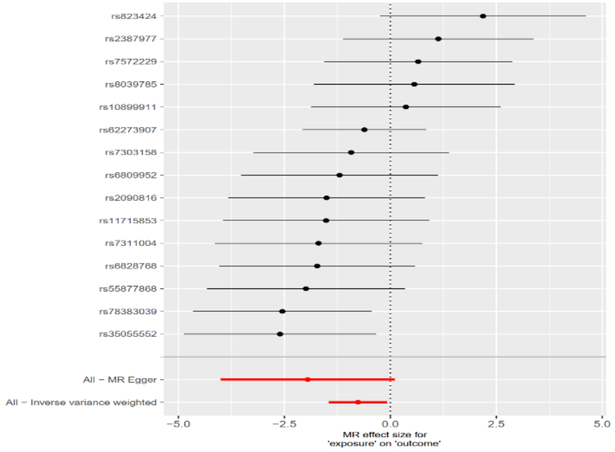


order.Enterobacteriales.id.3468

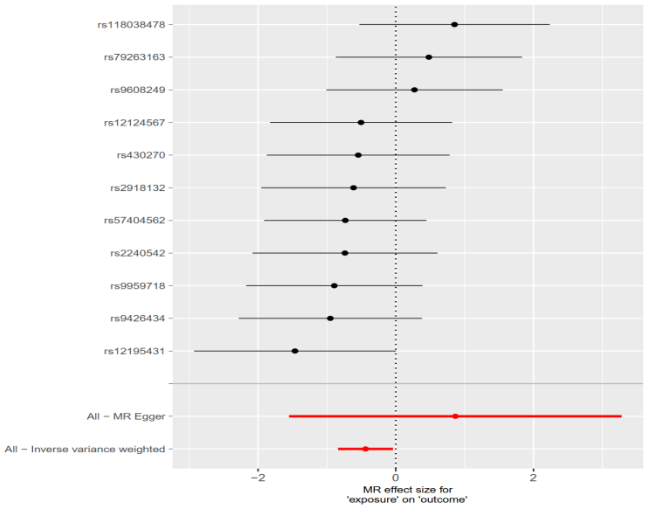


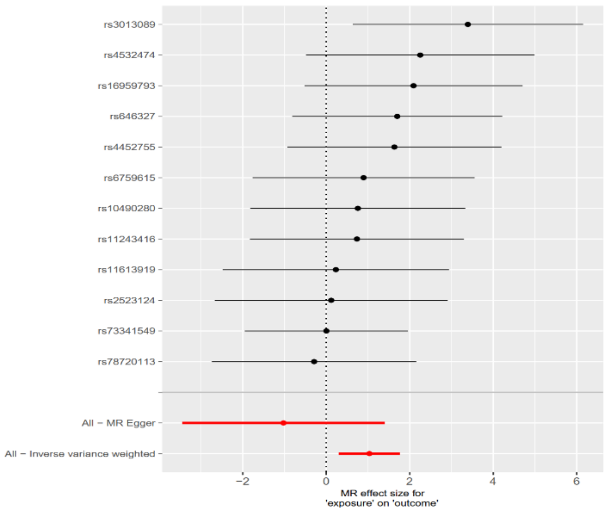


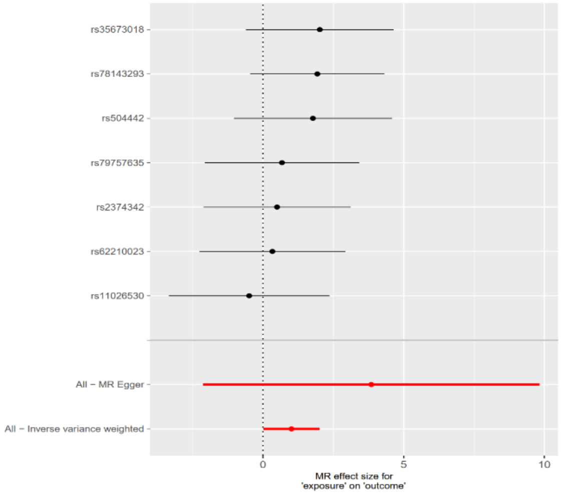


## order.Verrucomicrobiales.id.4030

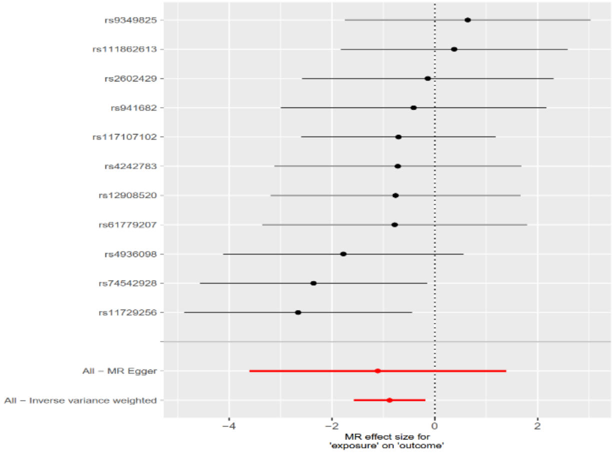


## phylum.Cyanobacteria.id.1500

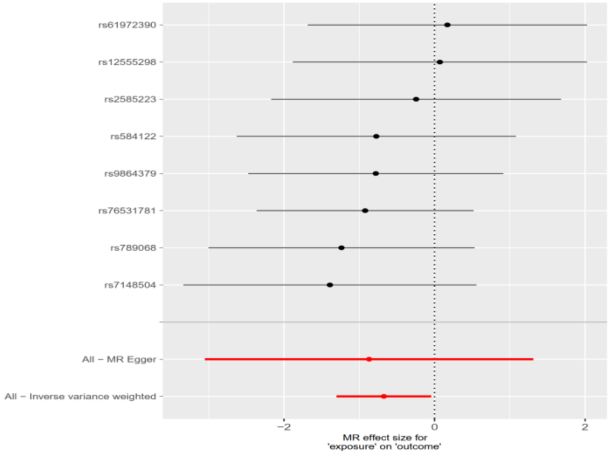

Supplement: Supplementary file 2 [file medi-103-e39677-s002.pdf]
